# Etiology-specific variation in survival following non-traumatic spinal cord injury: a causal inference approach using data from a population-based cohort

**DOI:** 10.1038/s41393-020-00554-9

**Published:** 2020-09-18

**Authors:** Anne Buzzell, Jonviea D. Chamberlain, Inge Eriks-Hoogland, Xavier Jordan, Martin Schubert, Marcel Zwahlen, Martin W. G. Brinkhof

**Affiliations:** 1 Swiss Paraplegic Research, Nottwil, Switzerland; 2 Department of Health Sciences and Medicine, University of Lucerne, Lucerne, Switzerland; 3 University of Bordeaux, Inserm, Bordeaux Population Health Research Center, Team VINTAGE, UMR1219 Bordeaux, France; 4 France Inserm, CIC1401-EC Bordeaux, France; 5 Swiss Paraplegic Centre, Nottwil, Switzerland; 6 Clinique Romande de Réadaptation, Sion, Switzerland; 7 Spinal Cord Injury Center, Balgrist University Hospital, Zurich, Switzerland; 8 Institute of Social and Preventative Medicine, University of Bern, Bern, Switzerland

**Keywords:** Cancer, Cardiovascular diseases, Epidemiology, Immunological disorders, Risk factors

## Abstract

**Study design:**

Observational, population-based cohort study.

**Objectives:**

To evaluate the origin and contribution to excess of survival differences following non-traumatic spinal cord injury (NTSCI) using etiology as proxy for variation in underlying health condition.

**Setting:**

Specialized rehabilitation centers in Switzerland.

**Methods:**

Medical record data collected by the Swiss Spinal Cord Injury cohort (SwiSCI) study were linked with mortality information from the Swiss National Cohort. Considering contemporary theory and empirical evidence, a directed acyclic graph (DAG) was developed to formally evaluate causal differences among NTSCI etiologies, relative to traumatic SCI (TSCI). Statistical inference was contingent on hazard ratios (HRs) and marginal survival differences, derived using flexible parametric modeling.

**Results:**

3643 individuals (NTSCI = 1357; TSCI = 2286) diagnosed with SCI between 1990 and 2011 were included, contributing a cumulative 41,344 person-years and 1323 deaths. Test statistics confirmed DAG-dataset consistency. As compared to TSCI, mortality was elevated in all NTSCI etiological groups; malignant etiologies had the highest HRs (10; 95% CI, 8.0 to 14) followed by infection (2.6; 1.8 to 3.6) and vascular (2.5; 2.0 to 3.2) etiology groups. At the attained age of 55, the estimated reduction in survival among non-malignant etiologies was 9.4% (5.8 to 13) at 5 years and 17% (11 to 23) at 20 years.

**Conclusions:**

Causal differences in survival among NTSCI etiological groups are likely a result of chronic variation in health conditions. This study supports the development of long-term interdisciplinary management and policy for individuals with NTSCI, specific to etiology.

## Introduction

Non-traumatic spinal cord injuries (NTSCI) are commonly associated with elevated mortality and reduced life expectancy [1–4]. The elevated risk of death is partly due to the impact of the neurological impairments on functioning and health, supported by the decline in survival with increasing level of spinal cord injury (SCI) severity. Yet, particularly for NTSCI, etiology-specific variation in chronic health conditions likely contribute to elevated mortality risk, mainly through acquired NTSCI etiologies; these include vascular disorders, neoplasms, and degenerative disc disorders, and infection. Moreover, the available clinical and epidemiological evidence regarding long-term functioning following diagnosis, as well as life-expectancy estimates suggest that even temporally-defined clinical events, such as infection or infarction, may be indicative of systemic and chronic differences in the health of persons with NTSCI of different etiology [2, 5, 6].

In order to define the scope for improving survival outcomes in NTSCI by adopting etiology-specific clinical management and health policy, population-based studies are needed that separate the mortality that is attributable to NTSCI etiology from lesion-related or demographic-attributable mortality. Such studies are timely in face of the growth in incidence and prevalence of NTSCI [2, 7]. We therefore used longitudinal data from a Swiss population-based cohort study [8] and applied formal causal inference methodology [9–11] to infer the strength of support for the hypothesis that mortality differentials in NTSCI causally relate to etiology group. Using the causal estimates for mortality differentials, we also estimated the disparity in survival that was directly attributable to etiology-specific differences.

## Methods

### Study description and vital status

The present study used medical records data collected from the Swiss Spinal Cord cohort study (SwiSCI), which includes all SCI-specific rehabilitation centers in Switzerland [8]. Individuals aged 16 years or older that initiated first SCI-rehabilitation between 1990 and 2011 were included in the present study. SwiSCI does not include disorders such as multiple sclerosis. Vital status data were obtained with high reliability (85.5%) through probabilistic data linkage with the Swiss National Cohort (SNC) using birthdate, date of death (when available), age, sex, and geocoded address, further described in-depth elsewhere [4]. The present study uses vital status data that was updated until December 31, 2018.

### Data management

International Spinal Cord Society (ISCoS) guidelines were followed in the grouping of all variables [12, 13]. NTSCI etiologies were grouped according to the International SCI core datasets for non-traumatic spinal cord injury [13]. The etiological categories included were identified as “acquired abnormalities” within this core dataset, mainly from the second level of classification, which included: ‘degenerative disc disorder,’ ‘infection,’ ‘vascular disorder,’ ‘benign tumor,’ and ‘malignant tumor.’ NTSCIs originating from tumors ‘benign’ and ‘malignant’ were split at the third level of classification, following previous research that has identified strong survival differences between these groups [3, 5, 6]. NTSCIs that originated from metabolic disorders, inflammatory disease, radiation-related causes, and those of an unclear origin were grouped into an ‘other’ category (*n* = 98). The TSCI population in the present study is made up of four major etiological groups, namely ‘transport-related,’ ‘sports and leisure,’ ‘falls,’ and ‘other.’ The main analysis utilized TSCI etiologies resulting from ‘transport-related’ and ‘sports and leisure’ etiologies for reference to NTSCI, as these etiological groups are least likely to be linked to underlying health conditions [14]. In a further sensitivity analysis, TSCIs due to ‘falls’ and ‘other’ were additionally included. Lesion level and completeness were combined into a four-level variable representing SCI severity, namely ‘paraplegia, incomplete,’ ‘paraplegia, complete,’ ‘tetraplegia, incomplete,’ and ‘tetraplegia, complete.’ Paraplegia was defined as lesions between T1-S5.

### Directed acyclic graph: graphical causal model

Causal inference is a formal methodology to achieve strong inference in empirical research. Causality is deduced by postulating and testing explanations of reality, relative to their fit with the measured data [15, 16]. The present study facilitated causal inference through the application of a directed acyclic graph (DAG). A DAG is a type of causal diagram that graphically encodes the hypothesized relationships between variables [16]. DAGs formalize causal inference by distinct specification of the direct path between the exposure and outcome variables of interest, i.e., the pathway defining the causal question, as well as alternative paths of association, which potentially induce bias. The latter particularly concerns confounding and potential colliding pathways, which need accounting for in study design and statistical analysis, as to minimize the risk of biased inference. A DAG encodes conditional independency statements that can be verified statistically against the data. *DAGitty* (http://dagitty.net), an online-based platform was used to build, edit, and support DAG evaluation [17].

#### Defining DAG parameters

**Fig. 1:**
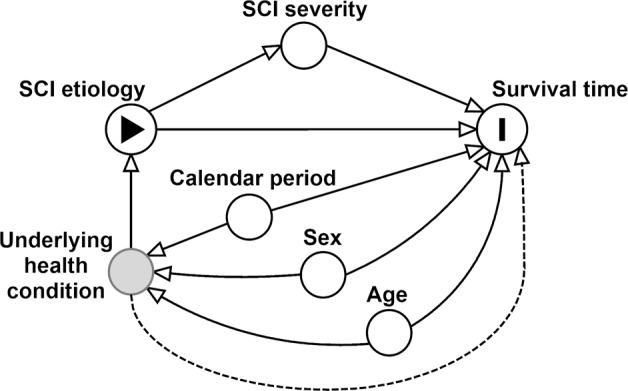
DAG of assumed causal relationship. Directed acyclic graph for the estimation of impact of underlying health conditions on mortality following NTSCI.

The effective DAG that was developed for the present study is presented in Fig. 1. ‘SCI etiology’ was defined as the exposure variable in the hypothesized DAG, which is used as a proxy for the assumed, but unmeasured variation in etiology-specific underlying health condition. NTSCI etiology was posited previously as a potential indicator for reduced survival, considering the differential life-expectancy seen between etiological groups [1, 5, 7]. Etiology-specific variation in “Underlying health conditions” was thus operationalized as the Latent parent of the exposure (http://dagitty.net for additional detail on graph terminology) [17]. ‘Survival time’ was defined as the outcome variable. The unidirectional arrow from the exposure to the outcome indicates the relationship for which causality was inferred in the present study. An additional arrow from the latent variable “underlying health conditions” to survival time was included—to account for the potential for unaccounted loss of survival directly caused by the underlying health condition.

An indirect causal influence is also represented by inclusion of a mediator, namely, ‘SCI severity’ (Fig. 1). This DAG therefore also hypothesizes that SCI etiology indirectly impacts mortality through SCI severity. This should be interpreted to mean that the NTSCI etiology, itself causes the SCI severity, which in turn has an impact on survival [5, 18, 19].

‘Age’ and ‘Sex’ define the confounding pathway, as they influence both the exposure ‘SCI etiology’, and the outcome ‘Survival time’ (Fig. 1). The reasoning for inclusion of these demographic factors as confounders is based on previous research in the field of SCI [7, 20]. ‘Calendar period’ defines a further potential confounding pathway, given that health survey and survival data from the Swiss general population indicate that the survival has steadily increased with calendar period, in addition to the declining risk of death following NTSCI [3, 19, 21].

This DAG guided all statistical analysis. In order to estimate survival differences across NTSCI etiological groups, we included survival data from persons with a TSCI etiology as a baseline reference. This baseline reference was determined to estimate the additional effect of underlying health conditions on survival, therefore the main analysis implemented the baseline TSCI due to ‘Transport’ and Sports/Leisure,’ as contemporary TSCI literature hypothesizes that TSCI due to ‘Falls’ or ‘Other’ may be linked to an underlying health condition [14]. It further facilitated the development of the minimal sufficient adjustment set, which identifies and characterizes the most critical variables that need to be accounted for in the comparative statistical models [16]. Using TSCI as a reference group additionally strengthened control for confounding pathways (through age and sex) in addition to the mediating pathway (through SCI severity) in the estimation of survival differences between NTSCI etiological groups—as well as relative to TSCI.

#### Determining conditional independences

Directed-separation, also known as d-separation is a set of criteria that describe how to interpret the statistical associations or the conditional independences that are implied by the DAG [22]. Testing these conditional independences provides an internal consistency check on whether the DAG is consistent with the effective dataset [23].

The R package *dagitty* implements the *DAGitty* web application and was used to evaluate the DAG-dataset consistency [23]. An internal check was applied to each conditional independence implied by the hypothesized DAG using logistic regression to calculate the root mean square error of approximation (RMSEA) [24]. Values <0.1 were considered consistent with our hypothesis [25].

### Statistical analysis

Statistical analyses were implemented using Stata software version 16.1 (College Station, TX).

Descriptive analyses report raw numbers and percentages stratified by etiological group, for age and length of stay, the mean and interquartile range are additionally reported.

Participant’s time-at-risk began with date of SCI diagnosis, with study start time beginning on the date of admission to first rehabilitation. Follow-up time was censored on the date of death, or the end of study (December 31, 2018), whichever came first. Kaplan–Meier estimates were used to graphically display crude survival according to etiology and to provide non-parametric estimates of median survival of the NTSCI with respect to the TSCI population.

Flexible parametric survival models were used to estimate hazard and survival functions between NTSCI and TSCI [26]. The Bayesian information criterion (BIC) was used to determine the minimal number of knots in the model. Age effects were accounted for by splitting follow-up time and apportioning quantities of follow-up time to the appropriate age categories. For example, if an individual was diagnosed with SCI at age 56 and died at the age of 64, this person would contribute 4 years of follow-up time to the age category “45–60 years” and 4 years to the “60–75 years” age group. Thus attained age was used as a timescale to better account for age differences. Cohort effects via calendar period were additionally accounted for through time-splitting [27], allotting participants to contribute time to different risk sets based on the time since diagnosis (1990–1999, 2000–2009, 2010–2018), during which they lived with their SCI [26]. Hazard ratios (HRs), with TSCI as the baseline reference were derived to estimate relative mortality. As a sensitivity analysis, HRs were estimated using TSCI from all causes (including TSCI due to ‘falls’ and ‘other’) as a baseline reference. Global significance testing derived *P*-values and 95% confidence intervals. *E*-values, which gauge the strength of an estimate considering the potential for unmeasured confounding, were additionally reported with lower CIs alongside HRs [28].

Major NTSCI etiological groups were identified using a post hoc analysis of Bonferroni-corrected pairwise comparisons of marginal linear predictions. The marginal survival differences in the NTSCI population compared to the reference group were estimated for calculation of absolute survival differences adjusted for sex, calendar period and SCI severity, using the attained age of 55, the average age of SCI diagnosis in this population, as the reference value [26]. Major etiological groups identified in the post hoc analysis (i.e. ‘non-malignant’ and ‘malignant’) were used to compare with the baseline reference group. An additional sensitivity analysis estimated the marginal survival differences in NTSCI using all cases of TSCI as the baseline.

## Results

The study population is described in Table 1. The present study includes 3643 individuals diagnosed with an SCI between 1990 and 2011, resulting in a cumulative 41,344 person-years and 1323 deaths. Of these individuals, 38% incurred an SCI from a non-traumatic origin. Vascular disorders were the most common NTSCI etiological group (27%) followed by degenerative disc disorders (25%). Across all NTSCI etiologies, females represented between 33 to 56% of the study population. The median age at diagnosis was 57 years in infection etiology, 58 years in benign tumors, 63 years in malignant tumors, 64 years in vascular etiology, and 65 in degenerative disc disorders. The proportion of individuals with tetraplegia varied between 13% (malignant tumor etiology) and 38% (infection etiology) among those with NTSCI (Table 1). Lesion completeness ranged between 4.1% in degenerative disc disorders up to 19% in NTSCIs due to malignant tumors. Significant differences were observed between etiological groups according to sex, age, SCI calendar period of onset, and SCI severity. Descriptive statistics stratified by TSCI etiology provided in Table S1.

**Table 1:** Characteristics of study participants by SCI etiology.

	Non-traumatic SCI, *n* (%)						
Person characteristics	Degenerative disc disorder	Infection	Vascular disorder	Benign tumor	Malignant tumor	Other	Traumatic SCI, *n* (%)
Overall survival characteristics							
*N* persons at risk (%)	337 (9.3)	126 (3.5)	368 (10)	129 (3.5)	304 (8.3)	93 (2.6)	2286 (100)
Person-years at risk	3275	1256	3435	1461	1006	827	30,085
*N* deaths	154	51	201	50	236	47	584
Sex							
Female	141 (42.1)	51 (40.8)	139 (37.8)	72 (55.8)	99 (32.6)	42 (45.2)	583 (25.5)
Male	194 (57.9)	74 (59.2)	229 (62.2)	57 (44.2)	205 (67.4)	51 (54.8)	1703 (74.5)
Age category							
16–30	9 (2.7)	15 (11.9)	17 (4.6)	16 (12.4)	14 (4.6)	12 (12.9)	700 (30.6)
31–45	48 (14.2)	21 (16.7)	33 (9.0)	19 (14.7)	43 (14.1)	24 (25.8)	575 (25.2)
45–60	85 (25.2)	40 (31.7)	87 (23.6)	36 (27.9)	72 (23.7)	23 (24.7)	459 (20.1)
61–75	117 (34.7)	32 (25.4)	166 (45.1)	37 (28.7)	127 (41.8)	22 (23.7)	360 (15.7)
75+	78 (23.2)	18 (14.3)	65 (17.7)	21 (16.3)	48 (15.8)	12 (12.9)	192 (8.4)
Calendar period of diagnosis							
1990–2000	58 (17.2)	39 (31.0)	103 (28.0)	31 (24.0)	87 (28.6)	19 (20.4)	903 (39.5)
2000–2011	279 (82.8)	87 (69.0)	265 (72.0)	98 (76.0)	217 (71.4)	74 (79.6)	1,383 (60.5)
SCI severity							
Paraplegia, incomplete	159 (50.5)	59 (50.9)	233 (65.5)	88 (71.5)	199 (68.9)	47 (53.4)	759 (34.2)
Paraplegia, complete	7 (2.2)	15 (12.9)	56 (15.7)	6 (4.9)	53 (18.3)	13 (14.8)	527 (23.7)
Tetraplegia, incomplete	146 (46.3)	36 (31.0)	57 (16.0)	29 (23.6)	33 (11.4)	26 (29.5)	709 (31.9)
Tetraplegia, complete	3 (1.0)	6 (5.2)	10 (2.8)	0 (0.0)	4 (1.4)	2 (2.3)	225 (10.1)

Overall *n* = 3643; NTSCI = 1357 with a cumulative of 41,344 person-years at risk; NTSCI = 11,260.

### Causal inference

Figure 1 illustrates the directed acyclic graph developed to hypothesize the relationship between underlying health conditions that precede the onset of NTSCI and mortality. The hypothesized DAG specified six conditional independences, or d-separation criteria, namely, (1) ‘Age at SCI is independent of sex’, (2) ‘Age is independent of SCI severity, given etiology’, (3) ‘Age is independent of calendar period of onset’, (4) ‘SCI severity is independent of sex, given etiology’, (5) ‘SCI severity is independent of calendar period of onset, given etiology’, and (6) ‘Sex is independent of calendar period of onset.’ RMSEA used for estimating DAG-dataset consistency was below 0.1 for all d-separation criteria (Table 2). For example, the RMSEA for independence testing of the d-separation ‘sex is independent of calendar period of onset’ was 0.03; in testing the independence of age and calendar period of onset, RMSEA was 0.08 (Table 2).

**Table 2:** DAG-dataset consistency evaluation.

Conditional independences	RMSEA
Age ⊥ Sex	0.0698
Age ⊥ SCI severity | SCI etiology	0.0246
Age ⊥ Calendar period of diagnosis	0.0820
SCI severity ⊥ Sex | SCI etiology	0.0627
SCI severity ⊥ Calendar period of diagnosis | SCI etiology	0.0342
Sex ⊥ Calendar period of diagnosis	0.0303

*RMSEA* root mean square error of approximation.

These estimates are reflective of a DAG-dataset consistency evaluation, where the conditional independences illustrated above DAG were checked with the dataset. Logistic regression was implemented to calculate RMSEA. All RMSEA values were lower than 0.1 for all conditional independence statements, which lends support to the current model. Reading example (line 2): “Age is independent of SCI Severity, conditional on Etiology”.

### Etiology-specific survival following NTSCI

**Fig. 2:**
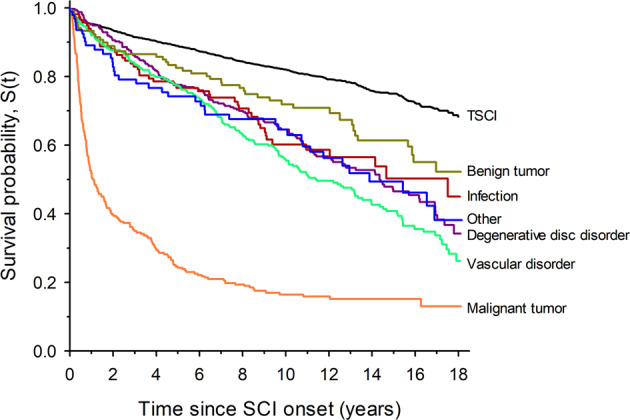
Kaplan–Meier survival curve for NTSCI by etiological group. Figure 2 illustrates the crude survival between etiological groups.

Crude Kaplan–Meier survival curves for individual SCI etiologies are shown in Fig. 2. Distinct differences are depicted between TSCI and individual NTSCI etiological groups, with malignant neoplasms indicating the sharpest decline in survival.

Multivariable HR estimates for mortality risk in NTSCI etiological groups (controlling for sex, age, calendar period of SCI onset, SCI severity) are shown in Table 3. HRs revealed strong differences in etiology compared to TSCI. For instance, the mortality risk was 2.5 (95% CI, 2.0 to 3.2) times greater in vascular-related NTSCIs compared to TSCI. Comparatively, mortality risk for NTSCIs due to benign tumors were 2.1 times greater, compared to TSCI (95% CI, 1.5 to 3.0), corresponding to an *E*-value of 3.6 (lower CI: 3.78). Moreover, a post hoc analysis of pairwise comparisons of marginal linear predictions identified three major etiological groups, namely, ‘a’: TSCI, ‘b’: ‘non-malignant etiologies followed by all ‘c’: ‘malignant neoplasms.’ This analysis confirmed that mortality was elevated for all NTSCI etiologies as compared to TSCI (Table 3). Furthermore, malignant tumors (‘c’) were additionally associated with markedly higher mortality than the remaining NTSCI etiology groups (‘b’). Effect sizes for covariates controlled for in the model (sex, attained age, calendar period of SCI, and SCI severity) all indicated an increased risk of mortality (Table S2). The sensitivity analysis including all TSCI etiologies (i.e., including categories ‘Falls’ and ‘Other’) confirmed these etiological differences in survival, albeit with slightly attenuated HRs (Table S3).

**Table 3:** Relative mortality risk: univariable and multivariable hazard ratios from FPM survival model.

Characteristics	Univariable estimates		Multivariable estimates^a^		
	Hazard ratio (95% CI)	*P-*value	Hazard ratio (95% CI)	*P-*value	*E-*value (lower 95% CI)
Etiology		<0.0001		<0.0001	
TSCI (baseline)	Reference		Reference	a	
Degenerative disc disorder	4.61 (3.70–5.75)		2.19 (1.70–2.82)	b	3.80 (2.79)
Infection	4.14 (3.02–5.69)		2.55 (1.83–3.55)	b	4.54 (3.06)
Vascular disorder	5.54 (4.49–6.82)		2.50 (1.96–3.20)	b	4.44 (3.33)
Other	5.06 (3.62–7.08)		3.42 (2.36–4.96)	b	6.30 (4.15)
Benign tumor	3.46 (2.52–4.75)		2.10 (1.48–2.99)	b	3.62 (2.32)
Malignant tumor	18.52 (14.89–23.04)		10.43 (7.99–13.62)	c	20.35 (15.46)

Letters a–c indicate group-level difference based on Bonferonni-corrected pairwise comparisons of marginal linear predictions in a post hoc analysis.

^a^The multivariable estimates are adjusting for sex, age, calendar period, and SCI severity.

**Fig. 3:**
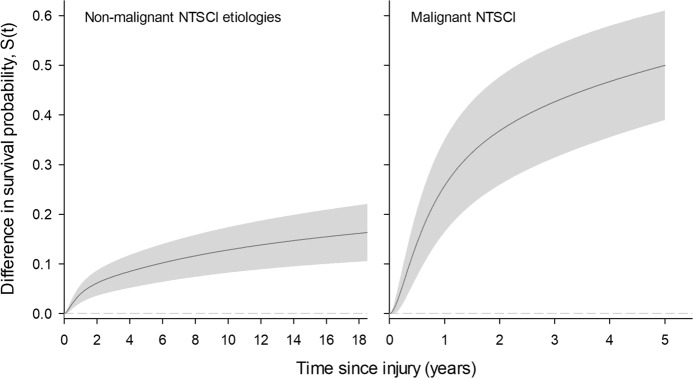
Marginal differences in absolute survival among NTSCI etiologies as compared to TSCI. All figures are adjusted for sex, age, calendar period of onset, and SCI severity. The figure on the left estimates the absolute survival difference in NTSCIs due to a non-malignant etiology, which included degenerative disc disorders, infection, vascular disorders, benign tumors, and others. The figure on the right illustrates survival differences in NTSCIs due to a malignant tumor. TSCIs (stemming from transport and sports/leisure-related injuries) was used as the reference group for both, with the gray area representing the 95% CI. Marginal survival differences are adjusted for sex, calendar period, and SCI severity, with the attained age of 55 was used as a reference value, as this was a common age of diagnosis in the present population. Survival was censored in cases of NTSCI due to a malignant etiology to only 5 years, following adjustment for baseline characteristics.

Marginal differences in survival according to major etiological groups are shown in Fig. 3. Adjusted survival differences using the average attained age of SCI onset, 55 years, as reference, depicted an increasing survival gap between non-malignant NTSCIs as compared to TSCI. For example, survival differences increase from 9.4% (5.8 to 13) at 5 years following diagnosis, to nearly 17% (11 to 23) after 20 years. The discrepancy was even more pronounced when comparing NTSCIs due to a malignant tumor to TSCI, with a reduction in survival diverging to 50% (CI = 39 to 61%) after 5 years following diagnosis. The sensitivity analysis including all TSCI etiologies gave similar results (Fig. S1).

## Discussion

This study supports inference that persons living with SCI of non-traumatic origin are inherently exposed to excess mortality that goes beyond mortality attributed to demographic factors, calendar period of onset, or SCI severity. In comparison with TSCI, regardless of etiology, individuals with an NTSCI had a consistently elevated mortality risk, excess mortality was greatest in those with a malignant tumor origin. These mortality differentials were unlikely due to unmeasured confounding given the magnitude of the *E*-values for the respective mortality hazard ratios. Furthermore, the stratified measure of NTSCI etiological groups demonstrated a widening gap in survival differences with time since injury, compared to traumatic SCI. Thus, evidence from this study supports the hypothesis that etiological differences in NTSCI are indicative of chronic variation in underlying health conditions.

The substantial reductions in absolute survival due to underlying health conditions indicate the need and potential for etiology-specific clinical care and policy. A recent analysis on the cause of death in the Swiss NTSCI population indicated cardiovascular disorders, neoplasms, and immunodeficiency as potential focus areas for the improvement of NTSCI-specific care [29]. NTSCIs due to benign or malignant tumors showed particularly higher mortality risk when compared to the reference group, which increased with time since injury. This evidence therefore has clear consequences for health policy and necessitates multidisciplinary care, co-management, and formally organized health-care guidelines. Transmural care approaches which aim for continuity of patient care and prevention could potentially serve as a model to bridge connections between rehabilitation physicians, family physicians, and homecare [30]. Therefore, results from this study can serve as a benchmark for guiding health-care systems and evidence-based policy. Implementation strategies of long-term treatment needs, which take into consideration the underlying health conditions in individual etiological groups, are essential. For example, a contemporary framework which considers the practical clinical management of individuals with NTSCI due to tumors is acronymed: NOMPRS [31]. NOMPRS indicates neurological status, oncologic status, medical comorbidities, pain, rehabilitation team, and social support. Similar structures and guidelines are needed for all NTSCI etiological groups with the addition of supporting long-term care. This would require inter-professional monitoring and care to manage individualized prevention strategies, based on the underlying health condition in coordination with SCI-specific care.

The major strength of this study is the formal implementation of a causal inference approach to derive estimate of the magnitude of reduced survival due to underlying health conditions following NTSCI within a high-income setting. Understanding the magnitude of survival inequities across NTSCI etiological groups in a formalized causal framework further provides a foundation for the development of improvement strategies. Owing to the design of SwiSCI, this study benefitted from a nationally representative sampling frame of individuals with SCI admitted to specialized rehabilitation. Furthermore, traditional and contemporary statistical methods were used to estimate differential survival, as exemplified through use of Kaplan–Meier survival curves as well as flexible parametric survival modeling, which permitted the evaluation of excess mortality, while accounting for sociodemographic and SCI-specific characteristics.

However, given that the present study relied on medical record data, an important limitation is the unavailability of potentially relevant confounders such as smoking and socioeconomic status; these factors, if included in the model may have potentially reduced the estimated effect of underlying health conditions on survival. Another issue is the potential selection bias regarding the admission to a specialized rehab hospital; this therefore limited the representativeness of this study to the SCI population in Switzerland that received specialized rehabilitation. Furthermore, individual health state in the present study was engendered by the latent variable ‘underlying health conditions’ in the causal pathway which precipitates SCI diagnosis. These underlying health conditions are therefore hypothesized to cause both SCI etiology and survival time. Our study strongly supported inference that underlying health conditions cause etiological-specific differences in survival. However, the pathways for clinical and biopsychosocial intervention require further investigation.

In addition, TSCI (due to Sports/Leisure, Transport) has not been proven to be an unbiased baseline reference as these individuals with TSCI may have had underlying health conditions which led to their injury. However, sensitivity analyses (which included all cases of TSCI, additionally due to falls and other causes) lent further support to the idea that underlying health conditions linked to NTSCI-specific etiology impacted survival to a greater extent following NTSCI. This study was further limited in its ability to discriminate SCI lesion level and severity, as the ‘International Standards for Neurological Classifications of Spinal Cord Injury’ score was not systematically measured until after the year 2001. Individuals with high and severe lesions (C1-C4 AIS A, B) often require respiratory assistance, which could impact the magnitude of the survival differential between etiological groups. Furthermore, length-time bias related to variant onset between etiological groups could have resulted in an underestimation of survival estimates, particularly for diseases with prolonged onset (e.g., spinal stenosis). However, we expect this would result in minimal bias on differential survival, given the amount of follow-up time in the present study. Finally, information on the specific type of cancer related to the onset of neoplastic NTSCIs was not available for further stratification.

The development and application of the DAG allowed for a transparent representation of the causal assumptions in this study and ultimately informed the methodological and statistical approach. The d-separation criterion via *P*-values, which link information on strength and sample size, RMSEA was used to test effect sizes. However, it is important to note that the check for DAG consistency with a particular set of data can never imply formal proof of the supposed association structure [22]. Therefore, international comparisons of survival after NTSCI with respect to etiology are needed to better inform the validity of the current model.

## Conclusion

The present study provides further evidence that sustained underlying health conditions impact mortality differentials following NTSCI, due to both non-malignant and malignant etiologies. The disparate survival following NTSCI indicates a need for targeted, long-term rehabilitation and prevention strategies that account for individual health conditions and implementations for interdisciplinary care. Furthermore, by defining the scope of the differential survival patterns specific to NTSCI etiology can potentially benefit service paradigms that aim to improve secondary prevention strategies through targeted clinical follow-up.

## Data archiving

Due to our commitment to SwiSCI study participants and their privacy, datasets generated during the current study are not made publicly available. The SwiSCI study center requires on behalf of the SwiSCI Study Group contact prior to any planned data usage (contact@swisci.ch).

## Supplementary information

Supplementary Tables and Figures

## References

[1] Ronen J, Goldin D, Bluvshtein V, Fishel B, Gelernter I, Catz A (2004). Survival after nontraumatic spinal cord lesions in Israel. Arch Phys Med Rehabil.

[2] New PW, Cripps RA, Bonne, Lee B (2014). Global maps of non-traumatic spinal cord injury epidemiology: towards a living data repository. Spinal Cord.

[3] Buzzell A, Chamberlain JD, Gmünder HP, Hug K, Jordan X, Schubert M (2019). Survival after non-traumatic spinal cord injury: evidence from a population-based rehabilitation cohort in Switzerland. Spinal Cord.

[4] Chamberlain JD, Gmünder HP, Hug K, Jordan X, Moser A, Schubert M (2019). Comparison of all-cause and cause-specific mortality of persons with traumatic spinal cord injuries to the general Swiss population: results from a national cohort study. Neuroepidemiology..

[5] Hernán MA (2004). A definition of causal effect for epidemiological research. J Epidemiol Community Health..

[6] New PW, Eriks-Hoogland I, Scivoletto G, Reeves RK, Townson A, Marshall R (2017). Important clinical rehabilitation principles unique to people with non-traumatic spinal cord dysfunction. Top Spinal Cord Inj Rehabil.

[7] Clark JM, Marshall R (2017). Nature of the non-traumatic spinal cord injury literature: a systematic review. Top Spinal Cord Inj Rehabil.

[8] Post MWM, Brinkhof MWG, von Elm E, Boldt C, Brach M, Fekete C (2011). Design of the Swiss spinal cord injury cohort study. Am J Phys Med Rehabilitation.

[9] Glymour MM. Using causal diagrams to understand common problems in social epidemiology. Methods in social epidemiology. San Francisco: John Wiley & Sons Inc. 2006. p. 387–422.

[10] Hernán MA (2004). A definition of causal effect for epidemiological research. J Epidemiol Community Health.

[11] Hernán MA (2018). The C-Word: scientific euphemisms do not improve causal inference from observational data. Am J Public Health..

[12] DeVivo M, Biering-Sørensen F, New P, Chen Y. Standardization of data analysis and reporting of results from the International Spinal Cord Injury Core Data Set. Spinal Cord. 2011;49:596–9. 10.1038/sc.2010.172. 10.1038/sc.2010.172 21135863

[13] Biering-Sørensen F, DeVivo MJ, Charlifue S, Chen Y, New PW, Noonan V (2017). International spinal cord injury core data set (version 2.0)—including standardization of reporting. Spinal Cord..

[14] Chamberlain JD, Gmünder HP, Hug K, Jordan X, Moser A, Schubert M (2018). Differential survival after traumatic spinal cord injury: evidence from a multi-center longitudinal cohort study in Switzerland. Spinal Cord..

[15] Platt JR (1964). Strong Inference. Science, New Series..

[16] Chamberlain JD, Brinkhof MWG (2019). Using strong inference to answer causal questions in spinal cord injury research. Spinal Cord.

[17] Textor J, Hardt J, Knüppel S (2011). DAGitty: a graphical tool for analyzing causal diagrams. Epidemiology..

[18] New PW, Guilcher SJT, Jaglal SB, Biering-Sørensen F, Noonan VK, Ho C (2017). Trends, challenges, and opportunities regarding research in non-traumatic spinal cord dysfunction. Topics in Spinal Cord Injury Rehabilitation..

[19] Buzzell A, Chamberlain JD, Eriks-Hoogland I, Hug K, Jordan X, Schubert M (2020). All-cause and cause-specific mortality following non-traumatic spinal cord injury: evidence from a population-based cohort study in Switzerland. Spinal Cord.

[20] Savic G, DeVivo MJ, Frankel HL, Jamous MA, Soni BM, Charlifue S (2017). Long-term survival after traumatic spinal cord injury: a 70-year British study. Spinal Cord.

[21] Remund A, Cullati S, Sieber S, Burton-Jeangros C, Oris M (2019). Longer and healthier lives for all? Successes and failures of a universal consumer-driven healthcare system, Switzerland, 1990–2014. Int J Public Health..

[22] Pearl J, Mackenzie D (2018). The book of why: the new science of cause and effect.

[23] Textor J, van der Zander B, Gilthorpe MS, Liskiewicz M, Ellison GT (2016). Robust causal inference using directed acyclic graphs: the R package ‘dagitty’. Int J Epidemiol.

[24] Weng H-Y, Hsueh Y-H, Messam LLM, Hertz-Picciotto I (2009). Methods of covariate selection: directed acyclic graphs and the change-in-estimate procedure. Am J Epidemiol.

[25] Thoemmes F, Rosseel Y, Textor J (2018). Local fit evaluation of structural equation models using graphical criteria. Psychological Methods.

[26] Roysten P, Lambert PC (2011). Flexible parametric survival analysis using stata: beyond the Cox model.

[27] Jann B. Stata tip 8: splitting time-span records with categorical time-varying covariates. In: Newton JH, Cox NJ, editors. Seventy-six stata tips, 2nd edn. College Station, Texas: Stata Press; 2009. p. 11–12.

[28] VanderWeele TJ, Ding P (2017). Sensitivity analysis in observational research: introducing the E-Value. Ann Intern Med.

[29] Brommer B, Engel O, Kopp MA, Watzlawick R, Müller S, Prüss H (2016). Spinal cord injury-induced immune deficiency syndrome enhances infection susceptibility dependent on lesion level. Brain..

[30] Smeenk FW, de Witte LP, Nooyen IW, Crebolder HF (2000). Effects of transmural care on coordination and continuity of care. Patient Educ Couns.

[31] New PW, Marshall R, Stubblefield MD, Scivoletto G (2017). Rehabilitation of people with spinal cord damage due to tumor: literature review, international survey and practical recommendations for optimizing their rehabilitation. J Spinal Cord Med.

