# Supplementary material for: Etiology-specific variation in survival following non-traumatic spinal cord injury: a causal inference approach using data from a population-based cohort
Source: Spinal Cord. 2020 Sep 18;59(3):257–65. doi: 10.1038/s41393-020-00554-9 (PMC7943420; doi:10.1038/s41393-020-00554-9)
Supplement: Supplementary file 1 — Supplementary Tables and Figures [file 41393_2020_554_MOESM1_ESM.docx]

**Buzzell A, Chamberlain JD, Eriks-Hoogland I, Jordan X, Schubert M, Zwahlen M, Brinkhof MWG, Etiology-specific variation in survival following non-traumatic spinal cord injury: a causal inference approach using data from a population-based cohort. Spinal Cord (2020)**

**Supplementary information:**

**Table S1: Description of TSCI participants across etiological group**

This table provides information on demographic, SCI characteristics and follow-up time for TSCI etiological groups

**Table S2. Relative mortality risk: Multivariable hazard ratios from covariates controlled for in the FPM survival model**

This table provides HRs for attained age, sex, calendar period, and SCI severity, which were included in the multivariable FPM survival model (Table 2)

**Table S3. Relative mortality risk comparison including all cases of TSCI**

This table provides a sensitivity analysis, which includes all cases of TSCI (transport, sports/leisure, falls, and other) for multivariable hazard ratios from an FPM survival model

**Figure S1. Marginal differences in absolute survival among NTSCI etiologies as compared to TSCI (with reference to all TSCI cases)**

This graph is analogous to Figure 3 in the main analysis and provides a sensitivity analysis to estimate marginal survival differences within NTSCI and TSCI including all cases of TSCI (transport, sport/leisure, falls, and other)

**Table S1:** Descriptive table of major TSCI groups

| **TSCI Etiology, n (%)** | **Transport** | | **Sports/Leisure** | **Falls** | **Other** |
| --- | --- | --- | --- | --- | --- |
|  |  |  |  |  |  |
| **Overall survival characteristics** | | | | | |
| N persons at risk (%) | 716 (29) | | 548 (22) | 878 (36.1) | 292 (12.0) |
| Person-years at risk | 10676 | | 7951 | 10179 | 3598 |
| Number of deaths | 118 | | 64 | 326 | 101 |
| **Sex** |  | |  |  |  |
| Female | 159 (22.2) | | 96 (17.5) | 287 (32.7) | 102 (34.9) |
| Male | 557 (77.8) | | 452 (82.5) | 591 (67.3) | 190 (65.1) |
| **Age category** |  | |  |  |  |
| 16-30 | 325 (45.4) | | 221 (40.3) | 155 (17.7) | 49 (16.8) |
| 31-45 | 188 (26.3) | | 168 (30.7) | 184 (21.0) | 76 (26.0) |
| 45-60 | 114 (15.9) | | 98 (17.9) | 207 (23.6) | 75 (25.7) |
| 61-75 | 60 (8.4) | | 56 (10.2) | 188 (21.4) | 72 (24.7) |
| 75+ | 29 (4.1) | | 5 (0.91) | 144 (16.4) | 20 (6.9) |
| **Calendar period of diagnosis** |  | |  |  |  |
| 1990-2000 | 336 (46.9) | | 191 (34.9) | 330 (37.6) | 118 (40.4) |
| 2000-2011 | 380 (53.1) | | 357 (65.2) | 548 (62.4) | 174 (59.6) |
| **SCI severity** |  | |  |  |  |
| Paraplegia, incomplete | | 172 (24.9) | 171 (32.1) | 277 (33.5) | 139 (49.8) |
| Paraplegia, complete | | 219 (31.7) | 80 (15.0) | 169 (20.5) | 59 (21.2) |
| Tetraplegia, incomplete | | 209 (30.2) | 181 (34.0) | 279 (33.8) | 40 (14.3) |
| Tetraplegia, complete | | 83 (12.0) | 67 (12.6) | 56 (6.8) | 19 (6.8) |

TSCI causes related to “Falls” and “Other” were excluded from main analyses as these etiological groups are potentially linked to underlying health conditions.

**Table S2:** Relative mortality risk: Multivariable hazard ratios from covariates controlled for in the FPM survival model (Table 2 in main analysis)

| **Characteristics** | **Multivariable estimates** | *P-value* |
| --- | --- | --- |
|  | Hazard Ratio (95% CI) |  |
| **Gender** |  | <0.0001 |
| Female | Reference |  |
| Male | 1.49 (1.28 - 1.74) |  |
| **Age group** |  | <0.0001 |
| 16-30 | Reference |  |
| 31-45 | 1.95 (1.16 - 3.28) |  |
| 46-60 | 4.18 (2.53 - 6.91) |  |
| 61-75 | 9.11 (5.49 - 15.10) |  |
| 76+ | 23.18 (13.89 - 38.69) |  |
| **Calendar period^1^** |  | 0.03 |
| 1990-1999 | Reference |  |
| 2000-2009 | 0.78 (0.60 - 1.02) |  |
| 2010-2018 | 0.70 (0.53 - 0.91) |  |
| **SCI severity** |  | <0.0001 |
| Paraplegia, incomplete | Reference |  |
| Paraplegia, complete | 1.45 (1.18 - 1.78) |  |
| Tetraplegia, incomplete | 1.07 (0.89 - 1.27) |  |
| Tetraplegia, complete | 3.00 (2.22 - 4.07) |  |

^1^Calendar period reflects the time since diagnosis that participants lived with their SCI.

**Table S3:** Relative mortality risk comparison (including all cases of TSCI)^1^

| **Characteristics** | **Multivariable estimates** | *P-value* |
| --- | --- | --- |
|  | Hazard Ratio (95% CI) |  |
| **Sex** |  | <0.0001 |
| Female | Reference |  |
| Male | 1.28 (1.13 - 1.45) |  |
| **Age Group** |  | <0.0001 |
| 16-30 | Reference |  |
| 31-45 | 2.46 (1.52 - 3.97) |  |
| 46-60 | 5.44 (3.44 - 8.61) |  |
| 61-75 | 13.61 (8.66 - 21.38) |  |
| 76+ | 38.02 (24.16 - 59.83) |  |
| **Calendar period** |  | <0.0001 |
| 1990-1999 | Reference |  |
| 2000-2009 | 0.89 (0.71 - 1.10) |  |
| 2010-2018 | 0.84 (0.67 - 1.05) |  |
| **SCI severity** |  | <0.0001 |
| Paraplegia, incomplete | Reference |  |
| Paraplegia, complete | 1.34 (1.14 - 1.58) |  |
| Tetraplegia, incomplete | 1.08 (0.93 - 1.24) |  |
| Tetraplegia, complete | 2.71 (2.16 - 3.39) |  |
| **Etiology** |  | <0.0001 |
| TSCI (baseline) | Reference |  |
| Degenerative disc disorder | 1.28 (1.06 - 1.54) |  |
| Infection | 1.53 (1.14 - 2.06) |  |
| Vascular disorder | 1.51 (1.27 - 1.80) |  |
| Other | 2.07 (1.48 - 2.90) |  |
| Benign tumor | 1.21 (0.89 - 1.64) |  |
| Malignant tumor | 6.30 (5.14 - 7.72) |  |

^1^ Cases of TSCI due to all causes (transport, sports/leisure, falls, and other) were used as a baseline reference in this model.

**Figure S1.** Marginal differences in absolute survival among NTSCI etiologies as compared to TSCI (with reference to all TSCI cases^1^)

**
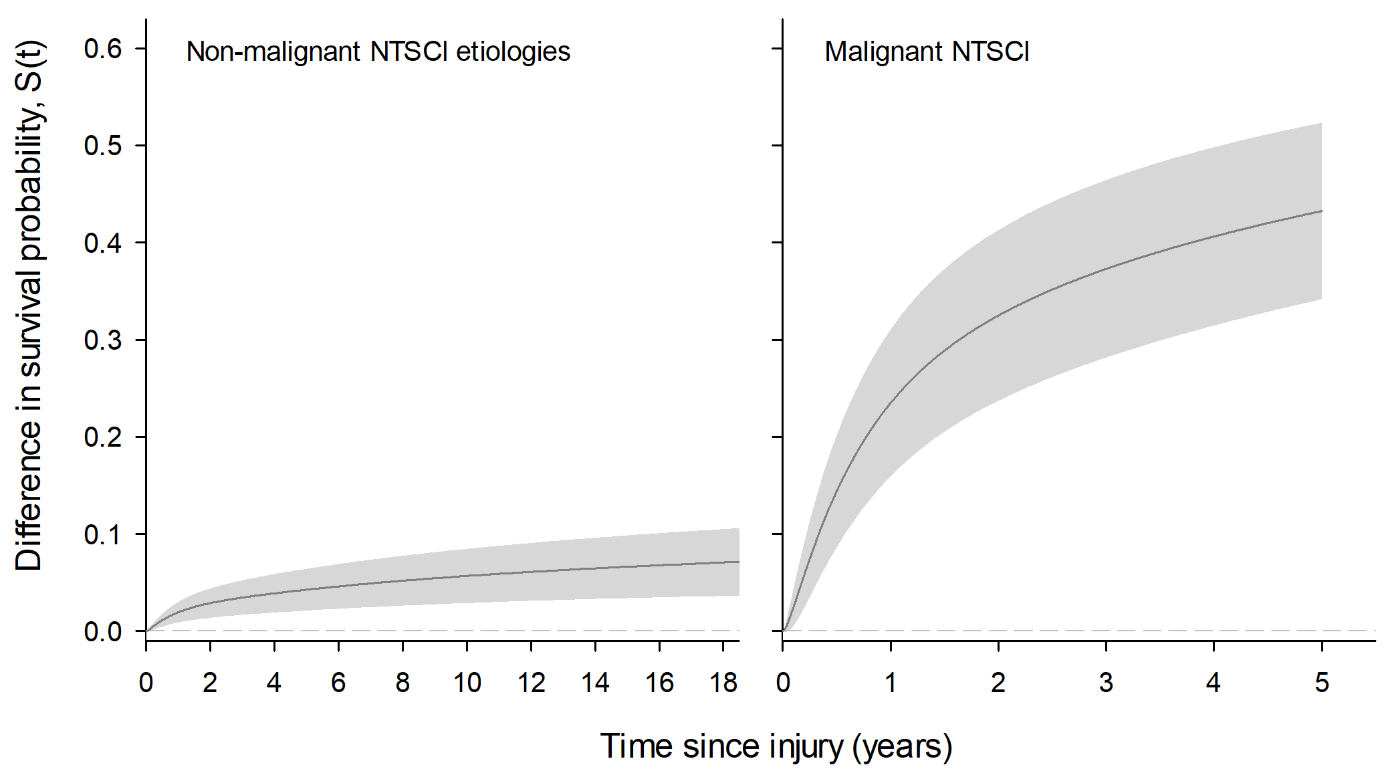
**

The figure on the left estimates the absolute survival difference in NTSCIs due to a non-malignant etiology, which included degenerative disc disorders, infection, vascular disorders, benign tumors and other. The figure on the right illustrates survival differences in NTSCIs due to a malignant tumor. All cases of TSCI (stemming from transport, sports/leisure, falls, and other)^1^ were used as the reference group for both, with the gray area representing the 95% CI.

Marginal survival differences are adjusted for sex, calendar period, and SCI severity, with the attained age of 55 was used as reference value, as this was a common age of diagnosis in the present population. Survival was censored in cases of NTSCI due to a malignant etiology to only 5 years, as survival in this group did not surpass this limit.
